# CMTM7 as a novel molecule of ATG14L-Beclin1-VPS34 complex enhances autophagy by Rab5 to regulate tumorigenicity

**DOI:** 10.1186/s12964-021-00720-3

**Published:** 2021-07-19

**Authors:** Baocai Liu, Yinliang Lu, Tingting Zhang, Xinyue Yu, Qian Wang, Yunbo Chi, Shunzi Jin, Guanghui Cheng

**Affiliations:** 1 Department of Radiation Oncology, China-Japan Union Hospital of Jilin University, Changchun, 130033 China; 2 NHC Key Laboratory of Radiobiology, School of Public Health, Jilin University, Changchun, 130021 China

**Keywords:** CMTM7, Autophagy, Rab5, ATG14L-Beclin1-VPS34, Tumorigenicity

## Abstract

**Background:**

CMTM7 is a tumor suppressor that positively regulates EGFR degradation by promoting Rab5 activation, and plays a vital role in tumor progression. Rab5 forms complexes with Beclin1 and VPS34, and acts in the early stage of autophagy. However, the affects of CMTM7 on autophagy and its mechanism are still unclear.

**Methods:**

The effect of CMTM7 on autophagy induction was confirmed by western blotting, confocal microscopy and transmission electron microscopy. Co-immunoprecipitation was used to analyse the interaction of CMTM7 with autophagy initiation complex and Rab5. The xenograft model in nude mice was used to elucidate the function of CMTM7 in tumorigenicity and autophagy in vivo.

**Results:**

In this study, we first demonstrated that CMTM7 facilitated the initiation of autophagosome formation, which consequently promoted the subsequent multistage process of autophagic flux, i.e. from autophagosome assembly till autolysosome formation and degradation. Confocal and co-immunoprecipitation showed that CMTM7 interacted with Rab5, VPS34, Beclin1, and ATG14L, but not with ULK1, UVRAG and LC3B. CMTM7 also increased the activity of ATG14L-linked VPS34 complex and its association with Rab5. Both in vitro and in vivo experiments demonstrated that knockdown of CMTM7 enhanced tumor growth by impairing autophagy.

**Conclusion:**

These findings highlighted the role of CMTM7 in the regulation of autophagy and tumorigenicity, revealing it as a novel molecule that is associated with the interaction of Rab5 and ATG14L-Beclin1-VPS34 complex.

**Video Abstract**

**Supplementary Information:**

The online version contains supplementary material available at 10.1186/s12964-021-00720-3.

## Background

Chemokine-like factor (CKLF)-like MARVEL transmembrane domain-containing proteins (CMTMs) is a family of proteins that structurally and functionally intermediate between classical chemokines and the transmembrane-4 superfamily [1–3]. In humans, the CMTMs are encoded by nine genes: *CKLF* and *CMTM1-8*. *CMTM7*, located on chromosome 3p22.3, is a tumor suppressor gene that is downregulated or absent in many cancer types [such as esophageal squamous cell carcinoma (ESCC) and non-small cell lung cancer (NSCLC)] due to promoter hypermethylation and loss of heterozygosity. CMTM7 inhibits the growth of ESCC and NSCLC cells by suppressing epidermal growth factor receptor-protein kinase B (EGFR-AKT) signaling [4, 5]. Moreover, knockdown of CMTM7 delays EGFR internalization and degradation by reducing Rab5 activation in lung cancer cells [5]. These data suggest that CMTM7 might act as a regulator of Rab5 activation, which in turn suppresses EGFR signaling and tumor progression.

Rab5 is a small GTPase that belongs to the Ras superfamily. It acts as a marker of early endosome and is necessary for the biogenesis of endolysosomal system [6]. Similar to other small GTPases, Rab5 cycles between active (GTP-bound) and inactive (GDP-bound) forms in order to regulate intracellular trafficking, such as EGFR internalization and degradation [7, 8]. Rab5 is also documented to be involved in autophagy, a membrane trafficking process that delivers intracellular contents destined for degradation into a double membrane structure, termed an autophagosome, which then fuses with lysosomes [9, 10].

Autophagy is like a double-edged sword that can either promote cancer cell survival or cell death, and could be determined by the level of autophagic flux [11]. Basal autophagic flux enhances cell survival, while elevated and prolonged autophagic flux promotes cell death. A complete autophagic flux comprises of the four successive steps, which are as follows: formation of phagophore, expansion and nucleation of phagophore into an autophagosome, maturation into amphisomes by fusing autophagosomes with endosomal compartments, and fusion with lysosomes to form autolysosomes, wherein the autophagosomal cargo is degraded and recycled [12].

Initiation of autophagosome biogenesis is done by UNC-51-like kinase (ULK) complex, and is negatively regulated by mammalian target of rapamycin complex 1 (mTORC1) [13]. Inactivation of mTORC1 leads to dephosphorylation, translocation and activation of ULK complex from the cytosol to the phagophore assembly site (PAS). During this, it recruits the autophagy-specific class III phosphatidylinositol (PtdIns) 3-kinase (PtdIns3K) complex, which primarily comprise of Beclin1 and VPS34, a class III phosphoinositide 3-kinase (PI 3-kinase) that converts phosphatidylinositol to phosphatidylinositol 3-phosphate. This is considered essential for initial nucleation and assembly of primary autophagosome membranes [14, 15]. Beclin1-VPS34 complex forms different complexes with varied binding partners, such as ATG14L and UVRAG [16, 17]. Mutual interaction between ATG14L and UVRAG with Beclin1 exhibits distinct functions of class III PI3K. ATG14L-linked VPS34 complex controls autophagy initiation and autophagosome formation [18, 19], while UVRAG complex is mainly involved in autophagosome maturation and endocytic fusion [20]. However, it is currently unclear as to what factors affect the activation of different VPS34-containing complexes.

Rab5 plays an important role in autophagy by inhibiting mTORC1 activation [21] or forming complex with Beclin1 and VPS34 [22, 23]. The autophagy process also involves few regulators of Rab5. For example, the class IA PI3-kinase p110β subunit maintains Rab5 activation and enhances Rab5-VPS34 interaction for promoting autophagy [24]. As a tumor suppressor and newly identified regulator of Rab5 activation [5], CMTM7 also plays a role in regulating the autophagy process. Therefore, we focused on the potential impact of CMTM7 on cell autophagy and involvement of Rab5 therein in this study.

## Materials and methods

### Cells, transfections and reagents

Human HeLa and A549 cells were purchased from the American Type Culture Collection (ATCC) and grown in RPMI-1640 medium supplemented with 10% fetal bovine serum (FBS, Thermo Fisher Scientific Inc., Waltham, MA USA). The stably transfected GFP-LC3B HeLa cell line was obtained as a gift from Yingyu Chen (Peking University, Beijing, China). Lentivirus-mediated shRNA knockdown of CMTM7 in A549 cells was done as described previously [5]. The cells were transfected with plasmids or small interfering RNAs (siRNAs) by using Lipofectamine 2000 reagent (Invitrogen, Grand Island, NY, USA). Constitutively active Rab5 and dominant-negative Rab5 plasmids were kindly provided by Yingyu Chen (Peking University, Beijing, China). Based on the pcDNA.3.1/myc-His(-)B (Invitrogen, V85520) plasmid, pcDB-CMTM7 plasmid and pcDB-CMTM7-myc-His plasmid were constructed, and abbreviated them as CMTM7 and CMTM7-myc, respectively. Along with these, mCherry-ATG5, mCherry-WIPI-1 GFP-RHEB^D60K^ and GFP-CMTM7 plasmids were also constructed in our laboratory. All plasmids were confirmed by DNA sequencing. siRNAs targeting Rab5 and VPS34 were designed and synthesized by GenePharma Co., Ltd. (Suzhou, China). The siRNA sequences were as follows: si-VPS34: 5′-GUGUGAUGAUAAGGAAUAUTT-3′, and negative control (si-NC): 5′-UUCUCCGAACGUGUCACGUTT-3′. Inhibition of autophagy was achieved by treating cells with 10 mM of 3-methyladenine (3-MA, Sigma-Aldrich, St. Louis, MO, USA) or 25 mM of chloroquine (CQ, Sigma-Aldrich, St. Louis, MO, USA). Other reagents used in this study were as follows: DAPI (Cell Signaling Technology, Inc., Beverly, MA, USA), Rapamycin (Selleckchem), and DMSO (Sigma-Aldrich, St. Louis, MO, USA).

### Co-immunoprecipitation and Western blot analysis

Co-immunoprecipitation assays were performed with control and CMTM7-myc-overexpressing HeLa cells. Cells were harvested after transfection for 36 h in a buffer containing 20 mM Tris–HCl, at pH 8.0, 150 mM NaCl, 2 mM EDTA, 10% glycerol, 0.5% Nonidet P-40, 1 mM dithiothreitol, 1 mM phenylmethylsulfonyl fluoride, 5 μg/ml leupeptin, 5 μg/ml aprotinin, 5 μg/ml pepstatin, and 1% protease inhibitor cocktail (Roche, Basel, Switzerland). Protein concentrations were determined by using BCA protein assays (Pierce, Rockford, IL, USA). Whole-cell lysates (1000 μg) were incubated with the indicated antibodies for 12 h at 4 °C and further incubated for another 2 h after adding pre-equilibrated protein-G Sepharose beads (GE, New York City, NY, USA). The sepharose beads were then washed thrice with the same buffer at 4 °C. The beads were collected by centrifugation, and then resuspended in 2 × SDS loading buffer and heated to 100 °C for 5 min. The whole-cell lysates were then fractionated using 10% or 15% SDS–PAGE gels and then electrotransferred onto polyvinylidene difluoride membranes (GE, New York City, NY, USA). Western blotting was performed according to the standard protocol as previously described [5], and the following antibodies were used: anti-Rab5 (Santa Cruz Biotechnology, Santa Cruz, CA, USA), anti-Beclin1, anti-VPS34, anti-ULK1, anti-ATG14L, anti-UVRAG, anti-myc, anti-GFP, anti-ATG5, anti-RPS6KB1, anti-phospho-RPS6KB1^Thr389^, anti-ATG12, anti-4E-BP1, anti-phospho-4E-BP1^Thr37/46^, anti-AMPKα, anti-phospho-AMPKα^Thr172^, anti-mTOR, anti-phospho-mTOR^Ser2448^, anti-ERK, anti-phospho-ERK^Thr202/Tyr204^ (all from Cell Signaling Technology, Inc., Beverly, MA, USA), anti-LC3B, anti-β-actin (Sigma-Aldrich, St. Louis, MO, USA), and anti-CMTM7 rabbit polyclonal antibody (Abcam, Cambridge, MA, USA). The protein bands were visualized by using a DyLight800-conjugated secondary antibody, and the signals were detected by an Odyssey Infrared Imager (LICOR Bioscience, Lincoln, NE, USA).

### Confocal microscopy

Cells were incubated with 50 nM LysoTracker Red (Invitrogen, Grand Island, NY, USA) for 20 min at 37 °C, washed with PBS, fixed and permeabilized with 3% paraformaldehyde containing 0.1% Triton X-100 for 30 min at 4 °C. They were then washed with PBS and stained with 4′,6-diamidino-2-phenylindole (DAPI, 3 mg/ml) (Sigma-Aldrich, St. Louis, MO, USA) for 10 min before being imaged with a TCS-SP laser-scanning confocal microscope (Leica Microsystems, Mannheim, Germany). The number of GFP-LC3B puncta per 20 cells was assessed, and statistical data were obtained from 3 independent experiments.

### Xenograft nude mice model

All protocols regarding animals were reviewed and approved by the institutional Animal Research Ethics Board of Jilin University. Female BALB/c nude mice (6 weeks old, weighing 20 g) were maintained in a germ-free environment in the animal facility. The tumorigenesis assay was performed as previously described [40]. Briefly, CMTM7 knockdown or control A549 cells were trypsinized and suspended in PBS for subcutaneous injection. A total of 1 × 10^6 ^cells in 100 μL PBS were injected subcutaneously into the right axilla of nude mice (four mice per group). Tumor diameters were measured with a caliper every 3 days, and tumor volumes were calculated according to length × width^2^ × 0.5. The mice were sacrificed after 4 weeks, when their tumors were dissected, weighed and fixed for immunohistochemistry and transmission electron microscopy analysis.

### Immunohistochemistry

Immunohistochemical analysis was performed on formalin-fixed, paraffin-embedded tumor tissues as previously described [40]. Briefly, tissue sections were dewaxed, rehydrated and placed in 10 mmol/L citrate buffer (pH 6.0) before being heated in a microwave oven for 10 min twice. Sections were incubated with 3% H_2_O_2_ for 10 min, washed with PBS, blocked with 10% normal goat serum for 30 min, and then incubated with anti-Ki67 (1:800) (Cell Signaling Technology, Inc., Beverly, MA, USA) at 4 °C overnight. After being washed, the sections were stained with reagents of the Catalyzed Signal Amplification System Kit (Dako, Glostrup, Denmark). Photos were acquired with a Nikon E800 camera.

### Transmission electron microscopy

A549 cells or tumor tissues were initially fixed in 0.1 M sodium phosphate buffer containing 3% glutaraldehyde (pH 7.4) at 4 °C and then fixed in 0.1 M sodium phosphate buffer containing 1% OsO4 (pH 7.2) for 2 h. The cells and tissues were dehydrated in an ascending series of acetone, embedded using an Ultracut microtome (LEICA ULTRACUT R, Bensheim, Germany) and sliced into 60 nm sections. Ultrathin sections were stained with uranyl acetate and lead citrate before being observed under a transmission electron microscope (TEM, H600IV electron microscope, HITACHI, Japan) at an accelerated voltage of 75 kV.

### In vitro PI(3)P ELISA

Total amount of PI(3)P was examined in a quantitative and competitive ELISA format assay according to the manufacture (Echelon Biosciences K-3000). Briefly, VPS34 containing complex was enriched by anti-ATG14L antibody and then mixed with phosphatidyl inositol (PI) substrates in an appropriate reaction buffer. After the PI3K reactions were complete and quenched, reaction products were diluted and added to the PI(3)P-coated microplate for competitive binding to a PI(3)P detector protein. The amount of PI(3)P detector protein bound to the plate was determined through colorimetric detection.

### Statistical analysis

The data are expressed as the mean ± s.d., and statistical analyses were performed using two-tailed Student's *t*-tests in Prism 5.0 (GraphPad Software, San Diego, CA, USA). Differences were considered significant if *P* < 0.05.

## Results

### Overexpression of CMTM7 increases autophagic flux

**Fig. 1:**
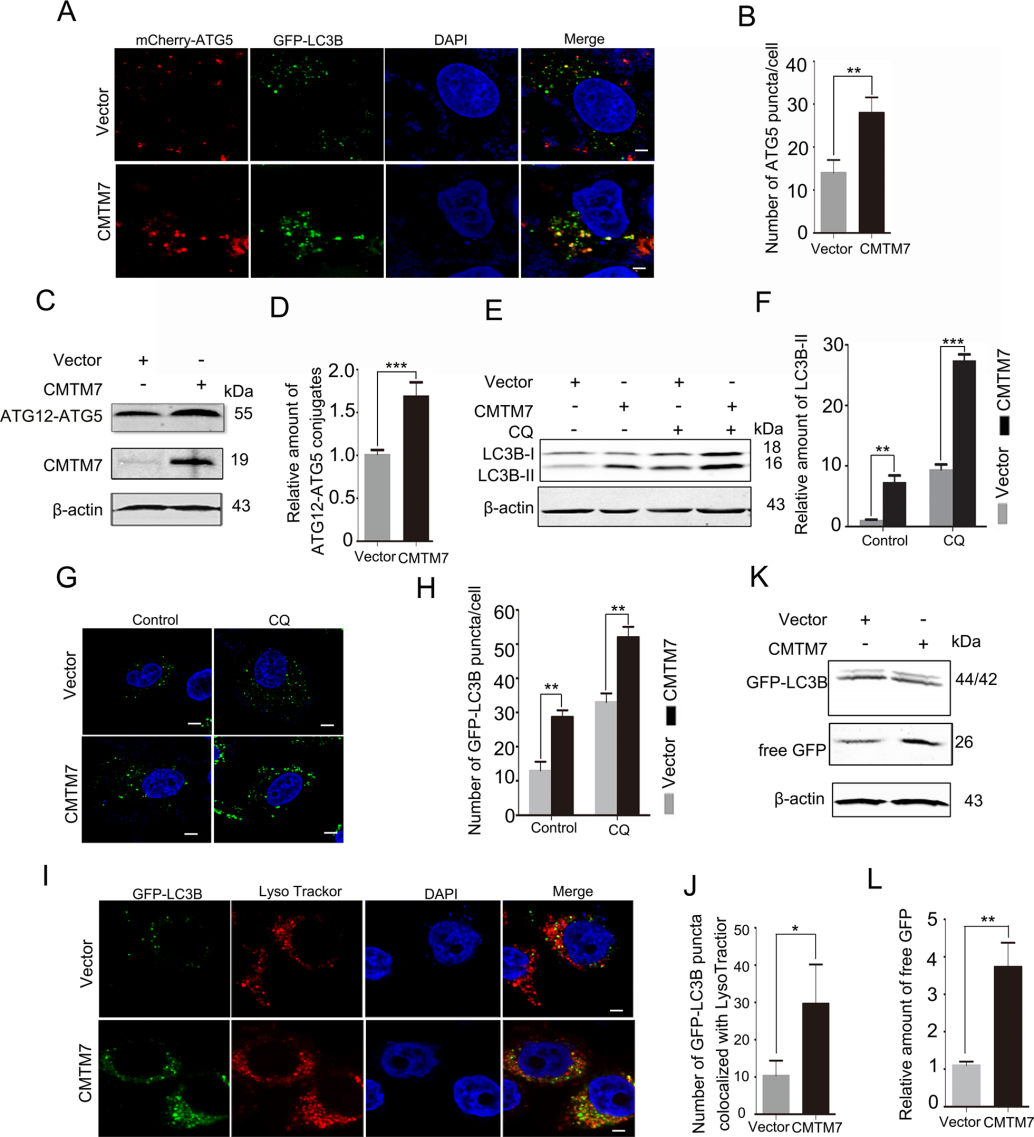
Overexpression of CMTM7 increases autophagic flux. **A** CMTM7 overexpression enhanced ATG5-labeled membrane structures. GFP-LC3B HeLa cells were co-transfected with CMTM7 and mCherry-ATG5 plasmids, and mCherry-ATG5 distribution at 24 h post-transfection was observed by confocal microscopy. Scale bars: 5 µm. **B** mCherry-ATG5 puncta per cell was quantified and expressed as means ± s.d. of at least 20 cells (**P < 0.01). **C** Western blotting of ATG12–ATG5 conjugation detecting by antibody against ATG12 in HeLa cells transfected with vector or CMTM7 plasmid. The intensity of the bands for ATG12–ATG5 conjugation protein was analyzed by ImageJ software (National Institutes of Health, Bethesda, Maryland, U.S.) and was normalized to the density of β-actin. The average relative grey density with s.d. from three independent experiments was shown in (**D**) (***P < 0.001). **E** HeLa cells were transfected with empty vector or CMTM7 plasmid, and after transfection for 24 h, the cells were treated with CQ (25 μM) or PBS as solvent control for 4 h. The level of LC3B was detected by Western blotting. β-actin was used as an internal standard. **F** Normalized densitometry data using ImageJ software according to the mean values of three independent experiments was presented as means ± s.d. (**P < 0.01, ***P < 0.001). **G** GFP-LC3B HeLa cells were treated as described in (**E**). Representative confocal microscopy images of GFP-LC3B distribution were shown. Scale bars: 5 µm. **H** GFP-LC3B puncta per cell were quantified and expressed as means ± s.d. of at least 20 cells (**P < 0.01). **I** GFP-LC3B HeLa cells were treated as described in (**E**), and representative fluorescence microscopy images of colocalization of GFP-LC3B with LysoTracker Red were shown. Scale bars: 5 µm. **J** The number of GFP-LC3B puncta co-localized with LysoTracker Red per cell was quantified and expressed as means ± s.d. of at least 20 cells (*P < 0.05). **K** GFP-LC3B HeLa cells were transfected with empty vector or CMTM7 plasmid, and after transfection for 24 h, the levels of free GFP were analyzed by Western blotting. The intensity of the bands for free GFP protein was analyzed by ImageJ software and normalized to the grey density of β-actin. The average relative grey density with s.d. from three independent experiments was shown in (**L**) (**P < 0.01)

Two ubiquitin-like conjugation systems are mostly involved in elongation and closure of phagophore. The first involves covalent conjugation of ubiquitin-like ATG12 to ATG5. The ATG12-ATG5 conjugates are localized onto the phagophore assembly site (PAS) and dissociate after autophagosome formation. The second involves conjugation of phosphatidylethanolamine (PE) to microtubule-associated protein 1 light chain 3-I (LC3-I, the non-lipidated form of LC3) to generate lipidated form of LC3, LC3-II, which in turn specifically targets ATG12-ATG5-associated phagophores by association with autophagosomes even after fusion with lysosomes [25, 26]. We first examined the impact of CMTM7 on the formation of phagophores by using mCherry-ATG5 as a labeling marker. As shown in Fig. 1A and B, overexpression of CMTM7 in HeLa cells stably overexpressing GFP-LC3B (GFP-LC3B HeLa cells) increased the number of ATG5-labeled membrane structures when compared to that in the vector transfected control cells. We further analyzed the levels of ATG12-ATG5 conjugates by Western blotting and the results showed an elevation in CMTM7-overexpressing HeLa cells (Fig. 1C, D), suggesting a promoting effect of CMTM7 on phagophore formation. The endogenous LC3B-II protein was then detected by Western blotting as a marker of autophagosome. Compared with vector control, LC3B-II level showed a remarkable increase in CMTM7-overexpressing HeLa cells (Fig. 1E, F). To identify whether this accumulation of LC3B-II occurred due to increased autophagosome formation or decreased autophagosome degradation, the lysosome inhibitor chloroquine (CQ) was used to prevent the fusion between autophagosomes and lysosomes or the degradation of autolysosomes by elevating the pH in lysosome and inactivating lysosomal acid hydrolases. As shown in Fig. 1E and F, still more LC3B-II was accumulated in CMTM7-overexpressing cells than in empty vector-transfected cells in the presence of CQ. This indicated that elevated LC3B-II levels driven by CMTM7 overexpression resulted due to increased autophagosome formation. The distribution of GFP-LC3B in GFP-LC3B HeLa cells was further monitored under a confocal microscopy. Consistent with Western blotting results, overexpression of CMTM7 increased the distribution of GFP-LC3B puncta when compared with control cells in the presence or absence of CQ (Fig. 1G, H). LysoTracker Red was used for labeling and tracking lysosomes in live cells. More GFP-LC3B puncta were observed in CMTM7-overexpressing cells than in control cells to co-localize with lysosomes (Fig. 1I, J), suggesting that overexpression of CMTM7 might facilitate fusion of autophagosomes and lysosomes. After delivering to lysosomes, the GFP-LC3B protein is cleaved and LC3B is rapidly degraded, while the GFP moiety remains relatively stable [27]. Thus, the level of free GFP corresponds to the autophagic flux. In GFP-LC3B HeLa cells, the free GFP level was upregulated by overexpression of CMTM7 when compared to vector control as illustrated by Western blotting analysis (Fig. 1K, L). Collectively, these data supported the promotion of CMTM7 on autophagic flux from autophagosome biogenesis till autolysosome formation and degradation.

### Knockdown of CMTM7 impairs autophagy

**Fig. 2:**
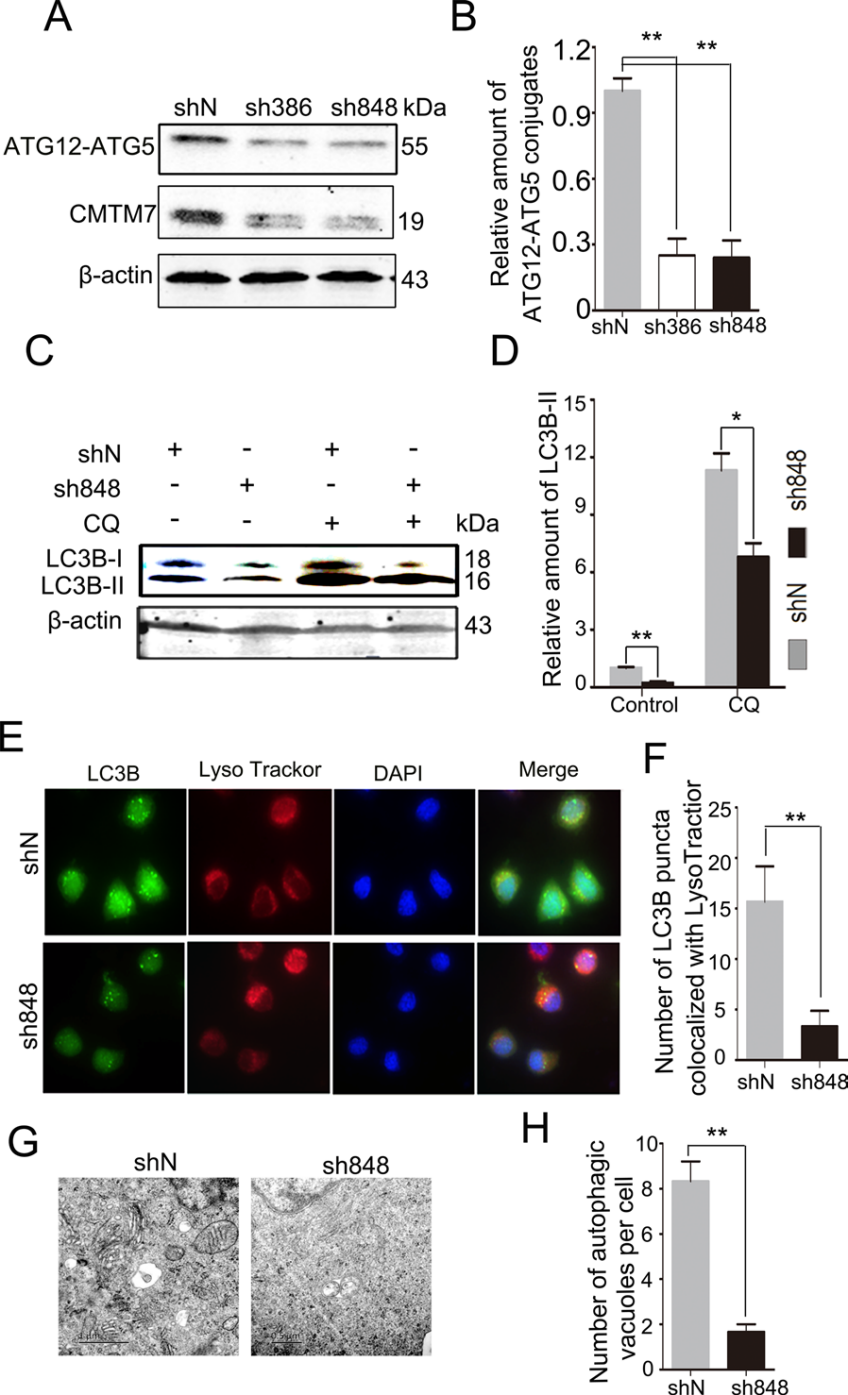
Knockdown of CMTM7 impairs autophagy. **A** Western blotting analysis of ATG12–ATG5 conjugation detecting by antibody against ATG12 in control and CMTM7 knockdown A549 cells. The intensity of the bands for ATG12–ATG5 conjugation protein was analyzed by ImageJ software and normalized to the grey density of β-actin. The average relative grey density with s.d. from three independent experiments was shown in (**B**) (**P < 0.01). **C** CMTM7 knockdown and control cells were treated with CQ (25 μM) or PBS as solvent control for 4 h. LC3B expression was detected by Western blotting, and β-actin was used as an internal standard. **D** Normalized densitometry data using ImageJ software according to the mean values of three independent experiments was presented as means ± s.d. (*P < 0.05, **P < 0.01). **E** Representative fluorescence microscopic images of endogenous LC3B co-localization with LysoTracker Red in CMTM7 knockdown and control cells. **F** The number of LC3B puncta co-localized with LysoTracker Red per cell was quantified and expressed as means ± s.d. of at least 20 cells (**P < 0.01). **G** Transmission electron microscopy analysis of autophagosome-like structures in CMTM7 knockdown and control cells. **H** The number of autophagic vacuoles per cell was quantified and expressed as means ± s.d. of at least 20 cells (**P < 0.01)

To determine the physiological effects of CMTM7 on autophagy regulation, experiments in stable CMTM7 knockdown A549 cells were conducted by using shRNA as identified in our previous study [5]. ATG12-ATG5 conjugates were examined by Western blot and found to be decreased in CMTM7 knockdown cells when compared with control cells (Fig. 2A, B). Similarly, LC3B-II was also downregulated by knockdown of CMTM7 and also CQ treatment did not reverse the downregulation trend (Fig. 2C, D). Decreased number of LC3B puncta was also observed in CMTM7 knockdown cells when compared with control cells, and so the cells were co-localized with LysoTracker Red-labeled lysosomes (Fig. 2E, F). Furthermore, transmission electron microscopy (TEM) was used to examine the ultrastructure of autophagic structures. These results showed that CMTM7 knockdown led to decreased autophagosome- or autolysosome-like structures than in the control cells (Fig. 2G, H). Taken together, these results suggested that CMTM7 facilitated cell autophagy.

### CMTM7 interacts with ATG14L-Beclin1-VPS34 complex

**Fig. 3:**
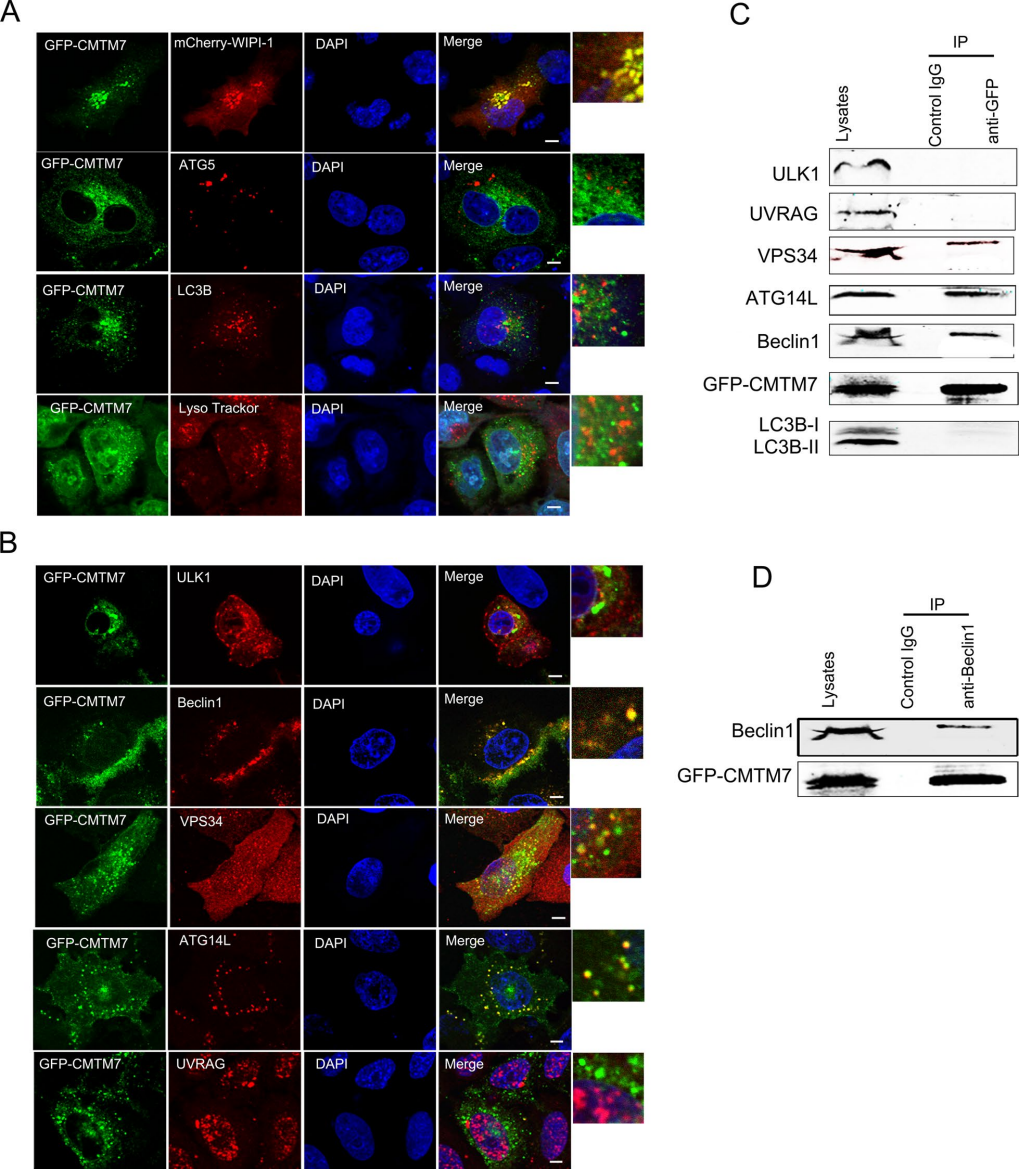
CMTM7 interacts with VPS34-Beclin1-ATG14L complex. **A** HeLa cells were co-transfected with CMTM7-GFP and mCherry-WIPI-1 for 24 h prior to fixation in 3% paraformaldehyde (PFA) and tested their co-localization, or only transfected with CMTM7-GFP and stained with antibodies against ATG5, LC3 or with LysoTracker Red. Scale bars: 5 µm. **B** HeLa cells were transfected with CMTM7-GFP for 24 h prior to fixation in 3% PFA and immunostained with antibodies against ULK1, Beclin1, VPS34, ATG14L or UVRAG. All autophagy associated markers are shown in red. Nuclei are visualized by DAPI (blue). Yellow color indicates colocalization. Scale bars: 5 µm. Control and CMTM7-GFP-overexpressing HeLa cells were subjected to immunoprecipitation with anti-GFP antibody (**C**), or anti-Beclin1 antibody (**D**), separated by SDS-PAGE and subjected to Western blotting. Immunoblots of the precipitates and the input were probed with indicated antibodies

According to the above data, CMTM7 promoted autophagic flux from autophagosome biogenesis to autolysosome formation and degradation, and immunofluorescence analyses were performed further to determine the intracellular distribution of GFP-CMTM7 in the regulation of autophagy. The results revealed that GFP-CMTM7 was co-localized with phagophore marker WIPI-1, but not with elongation marker ATG5, maturation marker LC3B, and lysosomal marker LysoTracker Red (Fig. 3A), indicating that CMTM7 might play an important role in the formation of phagophore. The current data suggest that ULK and Beclin1 complexes are important autophagic responders for phagophore. The results showed that GFP-CMTM7 had evident co-localization with Beclin1 rather than ULK1, which is a central component of ULK complex (Fig. 3B). Furthermore, GFP-CMTM7 was also co-localized with VPS34 and ATG14L, but not with the third key component of Beclin1 complex, UVRAG (Fig. 3B). We further tested the interaction between CMTM7 and Beclin1 by reciprocal co-immunoprecipitation (coIP) assays. GFP-CMTM7 plasmid was transfected into HeLa cells, which were lysed 48 h after transfection and then immunoprecipitated by anti-GFP and anti-Beclin1 antibodies, respectively. As shown in Fig. 3C and D, GFP-CMTM7 was co-precipitated with Beclin1 and vice versa, and GFP-CMTM7 also showed interaction with VPS34 and ATG14L, but not with ULK1, UVRAG and LC3B. These results suggested that CMTM7 was specially associated with ATG14L-Beclin1-VPS34 complex.

### CMTM7 increases the activity of ATG14L-linked VPS34 complex

**Fig. 4:**
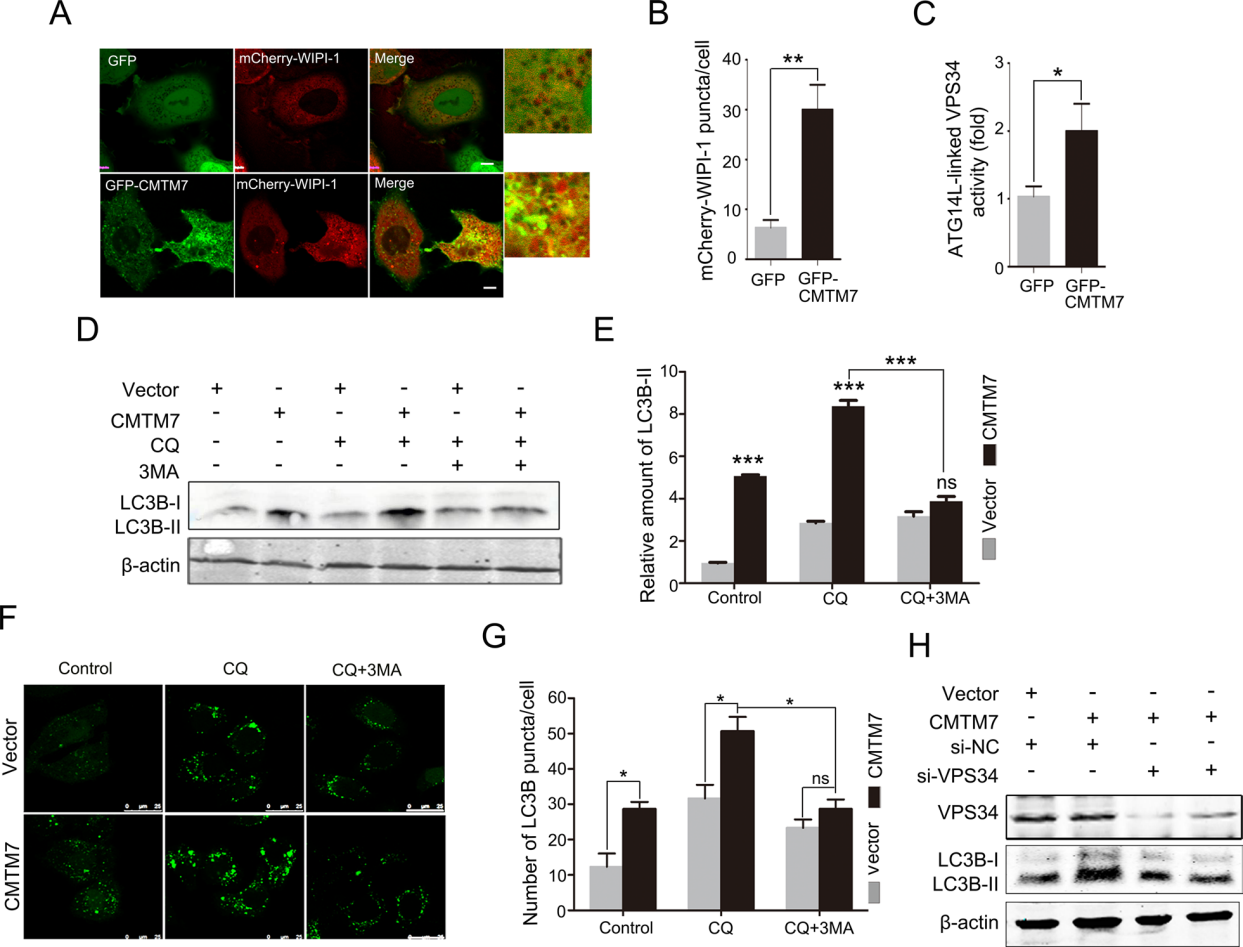
CMTM7 increases the activity of ATG14L-linked VPS34 complex. **A** HeLa cells were co-transfected with GFP-CMTM7 and mCherry-WIPI-1 plasmids, and after transfection for 24 h, the representative confocal microscopy images of mCherry-WIPI-1 distribution were shown. Scale bars: 5 µm. **B** mCherry-WIPI-1 puncta per cell were quantified by ImageJ software and normalized to the background of mCherry diffusion, and expressed as means ± s.d. of at least 20 cells (**P < 0.01). **C** Endogenous ATG14L was immunoprecipitated by control and GFP-CMTM7-overexpressing HeLa cells, and ATG14L-linked VPS34 kinase activity was measured by using the Class III PI3K ELISA Kit (*P < 0.05). **D** Western blotting analysis of LC3B expression in CMTM7 overexpressing and control cells treated with CQ (25 μM) for the final 4 h and/or 3-MA (10 mM) for the final 6 h was performed. β-actin was used as an internal standard. The intensity of the bands for LCB-II protein was analyzed by ImageJ software and normalized to the grey density of β-actin. The average relative grey density with s.d. from three independent experiments was shown in (**E**) (***P < 0.001; ns, not significant). **F** GFP-LC3B HeLa cells were transfected with empty vector or CMTM7 plasmid and treated as described in (**D**). Representative confocal microscopy images of GFP-LC3B distribution were presented. **G** GFP-LC3B puncta per cell were quantified and expressed as means ± s.d. of at least 20 cells (*P < 0.05; ns, not significant). **H** Control and CMTM7-overexpressing cells were transfected with control siRNA (si-NC) and siVPS34 (si-VPS34). Western blotting analysis of the levels of VPS34 and LC3B. β-actin was used as an internal standard

Beclin1-VPS34 complex phosphorylated phosphatidylinositol to generate phosphatidylinositol 3-phosphate (PI(3)P), promoting nucleation of phagophore and recruitment of autophagy downstream effectors, such as DFCP1 and WIPI-1 [28]. To investigate whether CMTM7 could regulate the activity of VPS34, the effect of CMTM7 on the major downstream effector of PI(3)P WIPI-1 was analyzed. As expected, CMTM7 overexpression in HeLa cells increased the number of punctuate structures of mCherry-WIPI-1 (Fig. 4A, B). To further demonstrate the effect of CMTM7 on VPS34 activity, the capability of PI(3)P production of VPS34 in CMTM7-overexpressing cells was determined by quantitative ELISA. Accordingly, CMTM7 overexpression increased the activity of ATG14L-linked VPS34 complexes (Fig. 4C). With regard to VPS34 containing complex, 3-methyladenine (3-MA), an inhibitor of VPS34, was used to assess its impact on CMTM7-induced autophagy. Elevated levels of LC3B-II in HeLa cells caused due to CMTM7 overexpression were significantly reversed by 3-MA treatment in the presence of CQ as examined by Western blotting (Fig. 4D, E). Confocal microscopy analysis results found that 3-MA significantly reduced the number of GFP-LC3B puncta than those increased in CMTM7-overexpressing HeLa cells with CQ treatment (Fig. 4F, G). A consistent result was obtained using siRNA to knockdown VPS34 in CMTM7-overexpressing HeLa cells (Fig. 4H). Collectively, these data indicated that CMTM7 can increase cell autophagy by enhancing the activity of VPS34.

### CMTM7 enhances the association of ATG14L-Beclin1-VPS34 complex with Rab5

**Fig. 5:**
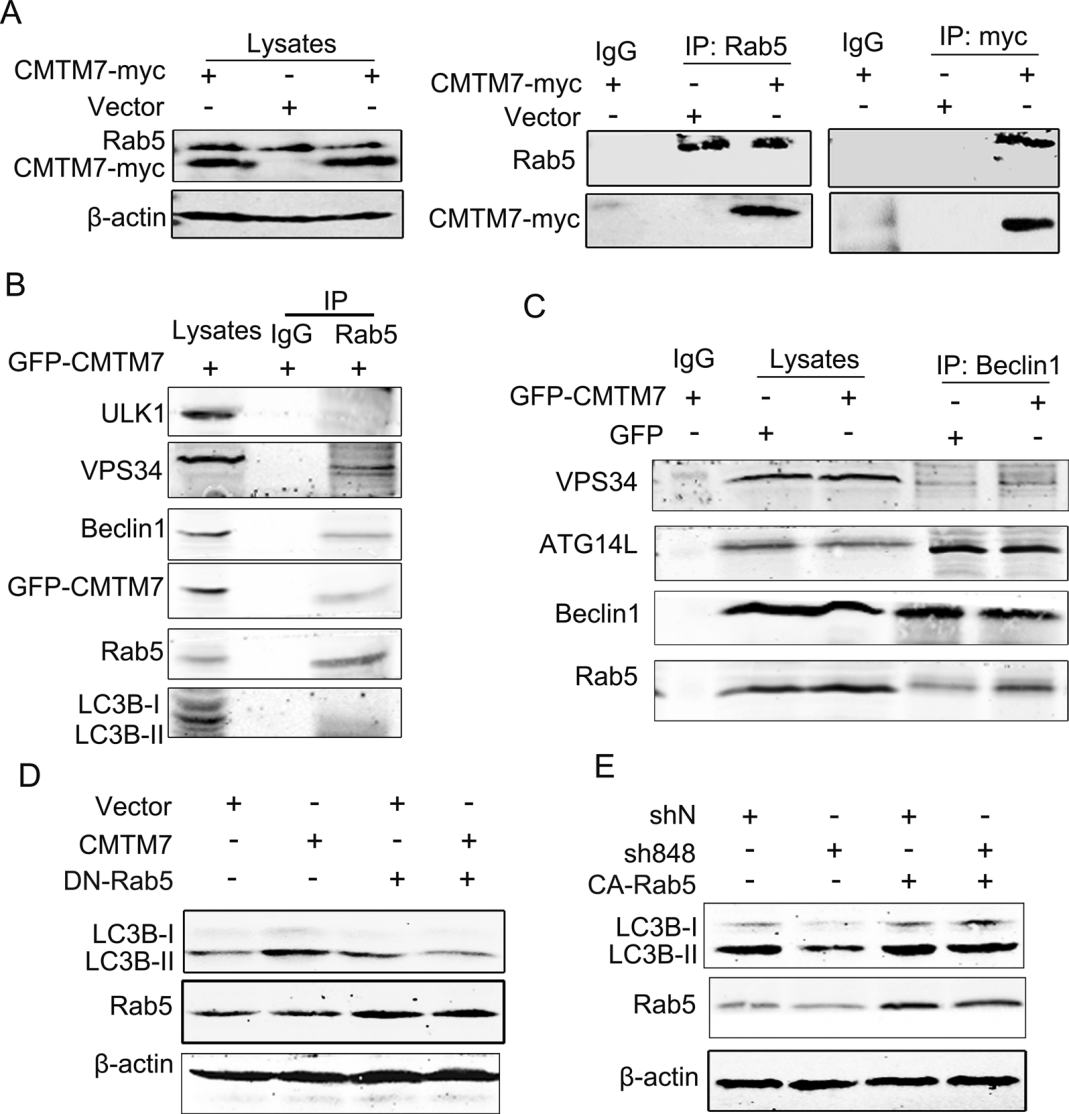
CMTM7 enhances the association of ATG14L-Beclin1-VPS34 complex with Rab5. **A** Control and CMTM7-myc-overexpressing HeLa cells were subjected to immunoprecipitation with anti-Rab5 antibody, anti-myc antibody or normal rabbit IgG, separated by SDS-PAGE and subjected to Western blotting. Immunoblots of the precipitates and the input were probed with the indicated antibodies. **B** Control and CMTM7-GFP-overexpressing HeLa cells were subjected to immunoprecipitation with anti-Rab5 antibody or normal rabbit IgG, separated by SDS-PAGE and then subjected to Western blotting. Immunoblots of the precipitates and the input were probed with the indicated antibodies. **C** Control and CMTM7-GFP-overexpressing HeLa cells were subjected to immunoprecipitation with anti-Beclin1 antibody, separated by SDS-PAGE and then subjected to Western blotting. Immunoblots of the precipitates and the input were probed with the indicated antibodies. **D** HeLa cells that were transiently transfected with CMTM7 were co-transfected with DN-Rab5. After 24 h post-transfection, the expression of LC3B was analyzed by Western blotting. β-actin was used as an internal standard. **E** CMTM7 knockdown and control cells were transfected with CA-Rab5. After post-transfection for 24 h, the expression of LC3B was analyzed by Western blotting. β-actin was used as an internal standard

The above results suggested that CMTM7 showed association with VPS34-Beclin1-ATG14L complex and enhanced VPS34 kinase activity. As reported previously that CMTM7 can increase the activity of Rab5, which interacts with VPS34 to regulate VPS34 activity and autophagy, we hypothesized that the promoting effects of CMTM7 on VPS34 activity might involve Rab5. Initially, the interaction between CMTM7 and Rab5 was assessed by coIP assays. CMTM7-myc plasmid was transfected into HeLa cells, which were lysed after transfection for 48 h and immunoprecipitated by anti-myc and anti-Rab5 antibodies, respectively. Western blotting showed that CMTM7-myc showed co-precipitation with Rab5 and vice versa (Fig. 5A), confirming the interaction of CMTM7 with Rab5. Furthermore, Rab5 also interacted with VPS34 and Beclin1, but not with ULK1 and LC3B (Fig. 5B), which was consistent with the reported results. This suggested that Beclin1-VPS34 complex contained Rab5 and CMTM7 simultaneously. CMTM7 further demonstrated increased association of Rab5 with ATG14L-Beclin1-VPS34 complex when using anti-Beclin1 antibody to immunoprecipitate ATG14L-Beclin1-VPS34 complex in GFP-CMTM7-overexpressing HeLa cells (Fig. 5C). To elucidate the effect of Rab5 on autophagy induced by CMTM7, ectopical expression of dominant-negative Rab5 (DN-Rab5) in CMTM7-overexpressing HeLa cells was performed and found that the increased formation of LC3B-II due to CMTM7 overexpression was hindered (Fig. 5D). In consistent with these, the CMTM7 knockdown-mediated LC3B-II decrease was recovered by constitutively active Rab5 (CA-Rab5) in CMTM7 knockdown A549 cells (Fig. 5E). Overall, these data supported the hypothesis that CMTM7 formed a complex with Rab5 and ATG14L-Beclin1-VPS34, exerting a positive influence on cell autophagy.

### mTOR is involved in CMTM7-induced autophagy

**Fig. 6:**
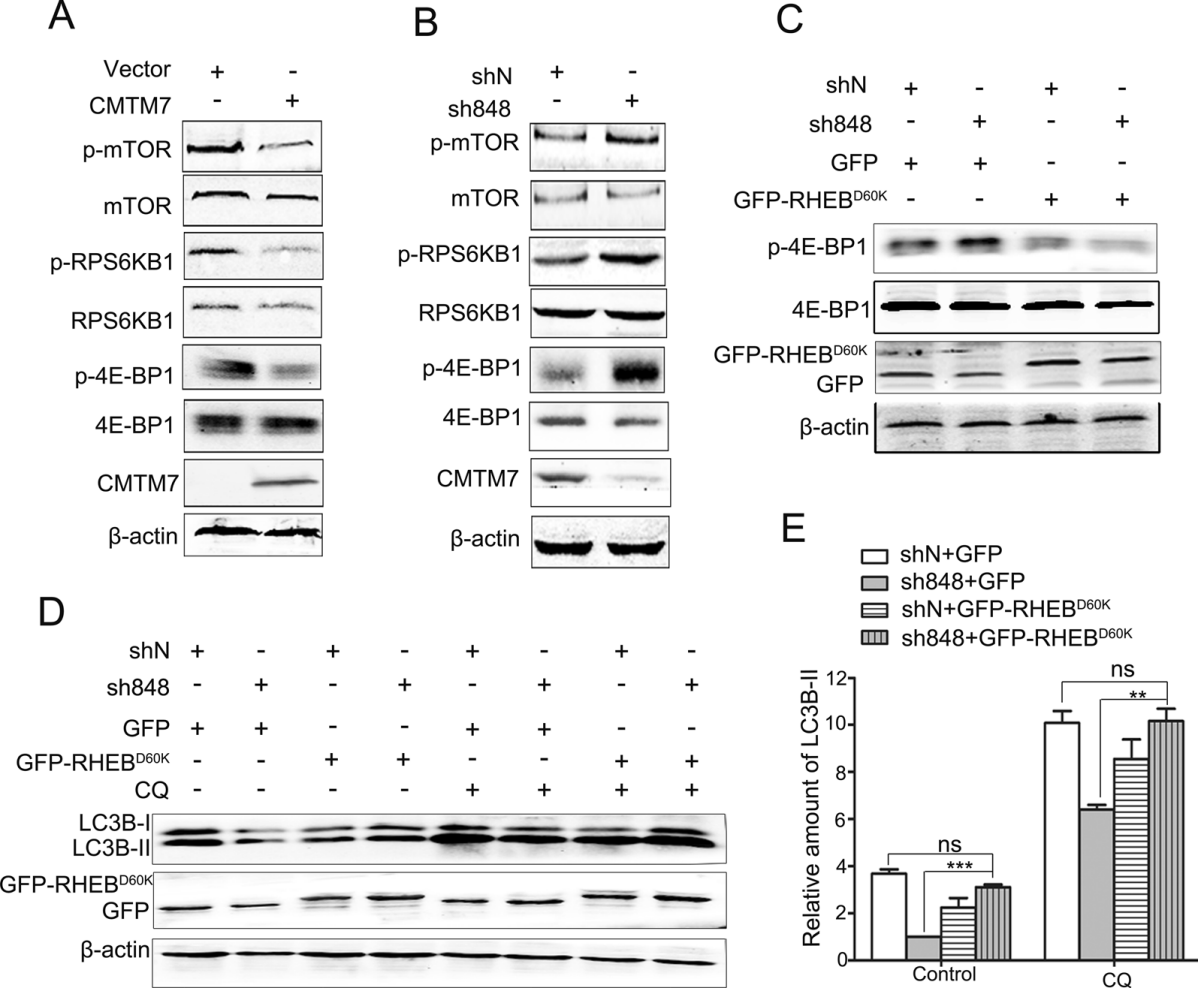
mTOR is involved in CMTM7-induced autophagy. **A** HeLa cells were transfected with empty vector or CMTM7 plasmid, and after transfection for 24 h, the total and phosphorylation levels of mTOR and its downstream signaling molecules RPS6KB1 and 4E-BP1 were analyzed by Western blotting. **B** The total and phosphorylation levels of mTOR and its downstream signaling molecules RPS6KB1 and 4E-BP1 in CMTM7 knockdown and control cells underwent Western blotting. **C** CMTM7 knockdown and control cells were transfected with vector expressing GFP-tagged RHEB-D60K mutant for 24 h, and the cell extracts were analyzed by Western blotting as indicated. **D** CMTM7 knockdown and control cells were transfected with vector expressing GFP-tagged RHEB-D60K mutant for 24 h, and then treated with CQ (25 μM) for 4 h, and then the expression of LC3B was analyzed by Western blotting. The intensity of the bands for LC3B-II protein was analyzed by ImageJ software and normalized to the grey density of β-actin. The average relative grey density presented as s.d. from three independent experiments was shown in (**E**) (**P < 0.05, ***P < 0.001; ns, not significant)

CMTM7 has been identified as an activator of Rab5 [5] that act in the early stage of autophagy by blocking mTORC1 activation [16]. We hypothesized that mTOR signaling might be involved in CMTM7-mediated autophagy. Initially, whether CMTM7 can affect the activation of mTOR was assessed, which showed an inverse correlation with autophagy induction. As illustrated by Western blotting, CMTM7 overexpression in HeLa cells downregulated the phosphorylation levels of mTOR at Ser2448 activation site. Correspondingly, phosphorylated RPS6KB1 and 4E-BP1, which are two downstream effectors of mTOR [29] were also decreased in CMTM7-overexpressing cells (Fig. 6A). Accordingly, phosphorylation of mTOR, RPS6KB1 and 4E-BP1 was increased in CMTM7 knockdown A549 cells (Fig. 6B). Furthermore, whether mTOR signaling is necessary for the autophagy suppression in CMTM7 knockdown A549 cells was evaluated by overexpressing an inactive form of RHEB^D60K^. This is used to inhibit the phosphorylation of RPS6KB1 and 4E-BP1 through inhibition of MTOR signaling [27]. As shown in Fig. 6C, the phosphorylation level of 4E-BP1 induced by CMTM7 knockdown was downregulated in RHEB^D60K^ overexpressing cells. Simultaneously, overexpression of RHEB^D60K^ recovered LC3B lipidation inhibited by CMTM7 knockdown (Fig. 6D, E). Taken together, these data suggested that the promoting effect of CMTM7 on autophagy inversely correlated with mTOR activity.

### CMTM7 knockdown-mediated autophagy suppression contributes to tumor cell proliferation

**Fig. 7:**
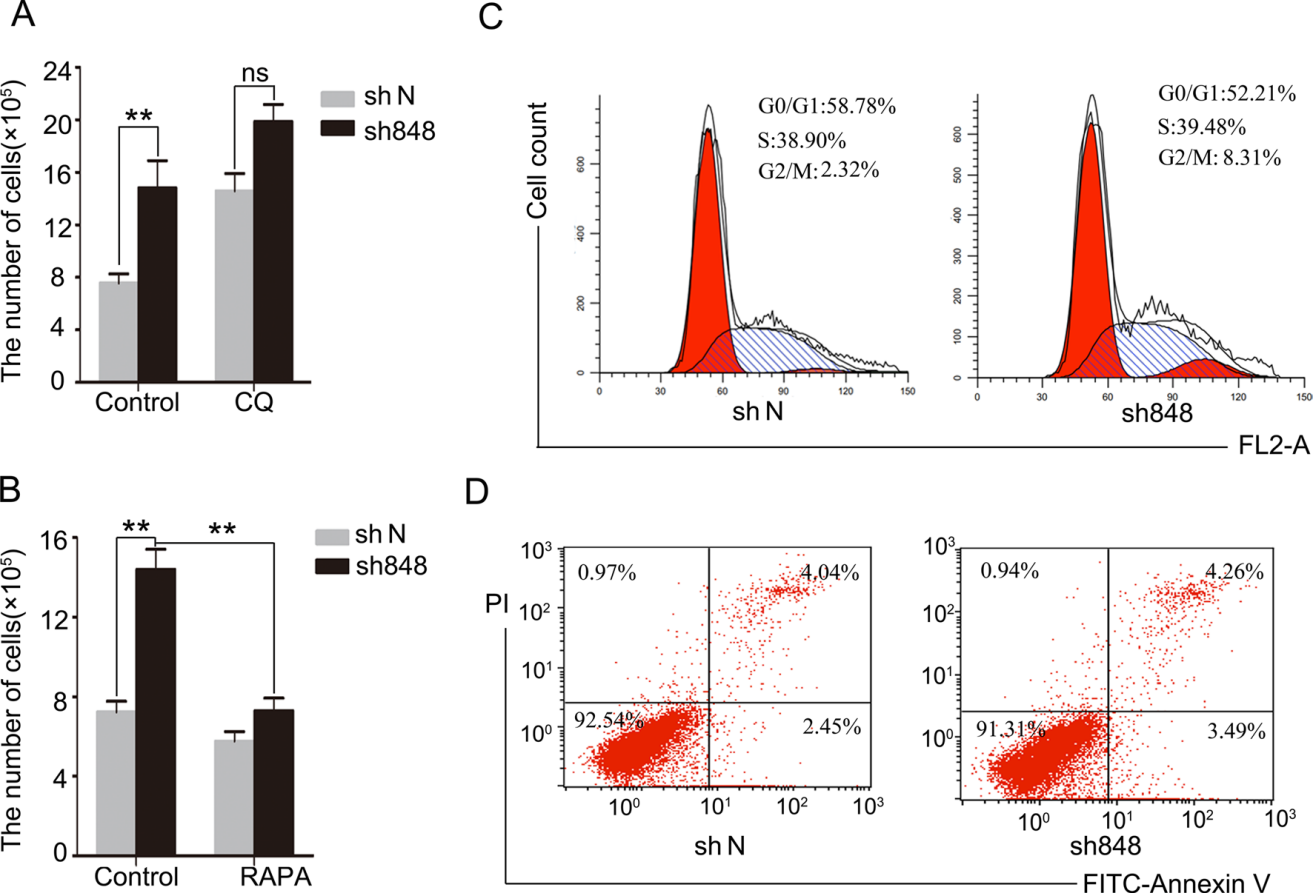
CMTM7 knockdown-mediated autophagy suppression contributes to tumor cell proliferation. **A** Control and CMTM7-knockdown A549 cells were plated at a cell density of 1.5 × 10^5^/mL in a 6-well plate, and 24 h after plating, the cells were treated with CQ (12.5 μM) or control reagent, and cultured for 2 days and counted using a blood counting chamber. Data are representative of three independent experiments, and expressed as means ± s.d. (**P < 0.01; ns, not significant). **B** Control and CMTM7-knockdown A549 cells were plated as described in (**A**), and treated with rapamycin (RAPA, 50 nM) or DMSO as a vehicle control, and cell number was counted after 2 days via using a blood counting chamber. Data are representative of three independent experiments, and expressed as means ± s.d. (**P < 0.01). **C** Cell cycle distribution was assayed by flow cytometry. Representative histograms (left) and the percentages of G0/G1, S and G2/M phase cells (right) were shown. **D** Apoptosis was measured by FITC-Annexin-V/PI staining and flow cytometry. Scatter plots are representative of three independent experiments

CMTM7 as a tumor suppressor demonstrated suppression of tumorigenicity [5] and promoted autophagy in vitro. To further assess whether the promoting effects of CMTM7 knockdown on tumor cell proliferation depended on autophagy in vitro, CMTM7 knockdown and control A549 cells with autophagy inhibitor CQ were treated and conducted cell counting assays. As shown in Fig. 7A, CQ treatment promoted cell growth in control A549 cells, which was similar to the level as CMTM7-knockdown cells, and suggested that CMTM7 knockdown might increase cell growth by depressing autophagy. As shown that autophagy inhibition that resulted from CMTM7 knockdown is dependent on mTOR activation, CMTM7 knockdown and control A549 cells were treated with mTOR inhibitor rapamycin and conducted cell counting assays. The results revealed that rapamycin reversed the promoting effects of tumor cell proliferation mediated by CMTM7 knockdown (Fig. 7B). Also CMTM7 knockdown showed no significant effects on cell cycle distribution (Fig. 7C) and cell apoptosis (Fig. 7D). Taken together, these results suggested that CMTM7 knockdown-mediated autophagy suppression contributed to tumor cell proliferation.

### Knockdown of CMTM7 attenuates autophagy and promotes tumorigenicity in vivo

**Fig. 8:**
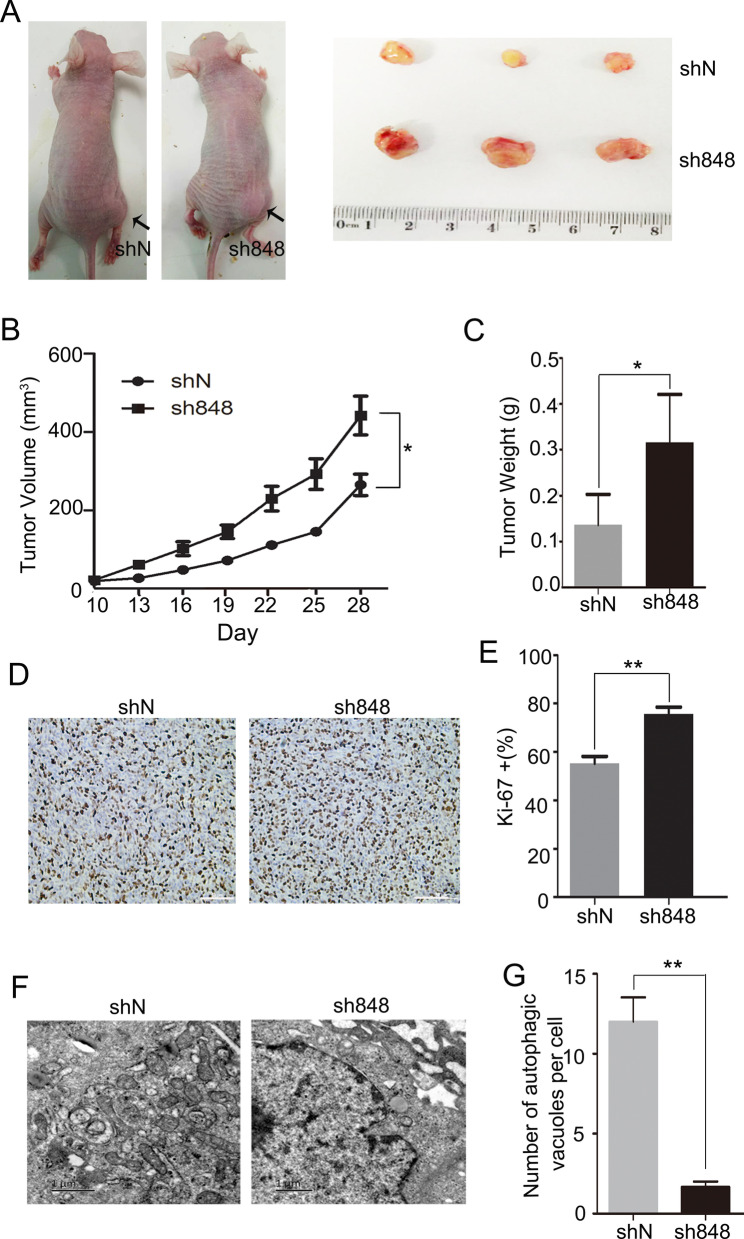
Effects of CMTM7 knockdown on tumorigenicity and autophagy in vivo. **A** Representative images of nude mice (left) indicated the morphology of tumors derived from negative control cells and CMTM7 knockdown cells. The tumors were dissected and then were photographed (right). The growth curve (**B**) and average weight (**C**) of CMTM7 knockdown tumors versus the control tumors were expressed as means ± s.d. for each group (n = 4) (*P < 0.05). **D** Immunohistochemistry for Ki-67 of representative areas from CMTM7 knockdown tumor and control tumor. Scale bars: 50 µm. **E** Expression of Ki-67 was quantified by the percentage of Ki-67 positive cells to the total cells. Data are presented as means ± s.d. (**P < 0.01). **F** Transmission electron microscopy analysis of autophagosome- or autolysosome-like structures in the tumors of mice inoculated with control and CMTM7 knockdown A549 cells. **G** The number of autophagic vacuoles per cell was quantified and expressed as means ± s.d. of at least 20 cells (**P < 0.01)

To confirm the promoting role of CMTM7 in autophagy and its tumor-suppressor function in vivo, CMTM7 knockdown and control A549 cells were subcutaneously injected into the right axilla of BALB/c nude mice. On day 28 after inoculation, the mice were euthanized and their tumors were removed and then were photographed (Fig. 8A). The tumor volumes and weights from CMTM7 knockdown group were significantly larger than those of the control group (Fig. 8B, C). Furthermore, immunohistochemical analysis demonstrated higher proportion of Ki-67-positive cells in CMTM7 knockdown tumors than in controls (Fig. 8D, E), suggesting that CMTM7 knockdown promoted cell proliferation and tumorigenicity in A549 cells. Consistent with the in vitro results, fewer autophagosome- or autolysosome-like structures were observed by TEM in the tumors of mice inoculated with CMTM7 knockdown cells than in control group (Fig. 8F, G). These data confirmed that CMTM7 increased cell autophagy and exhibited antitumor activity in vivo.

## Discussion

CMTM7 belongs to CMTM family of proteins, and comprises of a MARVEL domain. The MARVEL domain-containing proteins participate in a variety of cellular processes, such as tumorigenesis and transport vesicle biogenesis [30, 31]. Our previous work revealed that CMTM7 co-localizes with Rab5 on early endosomes and knockdown of CMTM7 increases tumorigenicity of lung cancer cells and EGFR-AKT signaling by reducing the activation of Rab5 [5], indicating that CMTM7 is involved in regulating intracellular membrane trafficking such as endocytosis and early endosome fusion. As Rab5 is a regulator of autophagy, it can be hypothesized that CMTM7 might also contribute to this vacuolar degradative process.

It has been demonstrated that several tumor suppressor proteins, such as BH3-only proteins, DAPK1, PTEN, TSC1 and TSC2, can induce autophagy [32, 33]. Our results showed that tumor suppressor CMTM7 interacted with the essential autophagy protein Beclin1 to facilitate the multistage process of autophagic flux, which was evidenced by the elevated levels of markers during different stages of autophagosome biogenesis, such as ATG12-ATG5 conjugates and LC3B-II, increased number of autophagosomes and their fusion with lysosomes, and accumulated free GFP released from GFP-LC3B protein by autolysosome degradation. However, it is worth noting that SQSTM1/p62 is well-known as an autophagy-related protein that degrades in autolysosome whose content therefore varies inversely with the activation level of autophagic flux, however, its expression is affected by many factors introduced in autophagy guidelines in detail [9]. In our experimental system, the effect of CMTM7 on SQSTM1/p62 is not obvious (data not shown), which needs further study. As elevated and prolonged autophagic flux inhibits tumorigenicity by regulating cell growth and autophagic cell death [34, 35], the promoting effect of CMTM7 on autophagy might contribute to its antitumor activity. In vitro experiments suggested that CMTM7-regulated cell growth was mediated by autophagy in CMTM7 knockdown A549 cells, but not by affecting cell cycle distribution and apoptosis. This was further verified by xenograft tumor model in nude mice, which showed that knockdown of CMTM7 impaired autophagy and accelerated tumorigenicity in vivo. Notably, other proteins that interact with Beclin1 also have demonstrated suppression of tumorigenicity by stimulating autophagy, such as ABHD5, UVRAG and Bif-1. ABHD5 promotes autophagy and inhibits tumorigenicity of colon cancer via interacting with and inhibiting the cleavage of Beclin1 by Caspase3 [36]. UVRAG interacts with Beclin1-Bcl-2-VPS34 complex, suppresses the proliferation and tumorigenicity of human colon cancer cells [37]. The deletion of Bif-1 in mice results in the development of spontaneous tumors [32]. These results suggest that CMTM7, a new interactor of Beclin1, can act as tumor suppressor by promoting autophagy.

It has been reported that by interacting with and activating PI3-kinase VPS34, Rab5 becomes a part of Beclin1-VPS34 complex, regulating ATG12-ATG5 conjugation in the early stages of autophagosome formation. Rab5 inhibition and loss of activity of VPS34 decreases ATG12-ATG5 conjugation and suppresses autophagy [22]. Our data showed that knockdown of CMTM7 reduced ATG12-ATG5 conjugates, which was in accordance with the inhibition of Rab5 activity caused by CMTM7 knockdown as previously reported [5] and overexpression of CMTM7 demonstrated an opposite effect. The confocal and co-IP assays discovered interaction of CMTM7 with Rab5 and ATG14L-Beclin1-VPS34 complex, but not with UVRAG. Some previous studies showed that ATG14L was not localized to membrane compartments labelled by early endosomal markers such as EEA1 and Rab5 [18, 41], however, we demonstrated the interaction of Rab5 and ATG14L-Beclin1-VPS34 complex in the CMTM7 overexpression HeLa cells, and found that CMTM7 increased the association of Rab5 with Beclin1 and elevated the activity of ATG14L-linked VPS34, suggesting that CMTM7 facilitated the formation of Rab5 and ATG14L-Beclin1-VPS34 complex. It has been reported that ATG14L-associated VPS34 complex is mainly responsible for autophagy initiation, while UVRAG-associated complex is necessary for autophagosome maturation and endosome fusion. Based on these results, we proposed that CMTM7 plays a crucial role in autophagy initiation by activating ATG14L-linked VPS34 complex. This consequently promotes multistage process of autophagic flux, from autophagosome assembly to autolysosome formation and degradation. However, the underlying molecular mechanism of how CMTM7 increases the interaction of Rab5 and ATG14L-Beclin1-VPS34 complex was not identified. As CMTM7 contains a MARVEL domain that is involved in the machinery of membrane apposition events, such as transportation of vesicle biogenesis and polarized membrane trafficking [30], we speculated that CMTM7 might promote the formation of Beclin1-VPS34 complex with Rab5 or other molecules through MARVEL domain, and this requires further validation.

On the other hand, overexpression of Rab5 at the initial stage by inhibiting mTOR activation induces autophagy. Besides, hyperactivation of endogenous Rab5 by overexpressing GAPex, which is a guanine nucleotide exchange factor for Rab5, inhibits mTORC1 as well [21]. The reason for this is that CMTM7 as a novel activator and interaction molecule of Rab5 might also have a similar capacity. Our experiment proved that the knockdown of CMTM7 resulted in elevated levels of phosphorylation of mTOR and its substrates RPS6KB1 and 4E-BP1. In addition, knockdown of CMTM7 increases EGFR-AKT signaling by reducing Rab5 activation [5]. Activated AKT-mediated inhibition of TSC1-TSC2 complex also activates mTOR signaling [9, 38, 39]. However, CMTM7 showed no significant influences on the phosphorylation of ERK [5] and AMPK (Additional file 1: Fig. S1), which are another two important molecules involved in the regulation of mTOR signaling and autophagy. Therefore, the inhibitory effect of CMTM7 on mTOR might be mediated by Rab5 either directly or indirectly.

Accumulating evidence indicate that CMTM7 acts a dual modulator for both class I PI3K and class III PI3K by completely different regulatory patterns. As reported previously, CMTM7 decreases class I PI3K activity by repressing EGFR internalization and trafficking. But CMTM7 increased the activity of ATG14L-linked class III PI3K in this study. Thus, CMTM7 might be considered to play a crucial role as a tumor suppressor by inversely regulating the two types of PI3K. Namely, on one hand, CMTM7 inhibits over-activation of class I PI3K signaling induced by growth factors, such as EGF, and on the other hand, CMTM7 promotes autophagic flux to suppress cell growth. Therefore, through different regulator mechanisms on two classes of PI3K, CMTM7 more effectively exerts tumor-suppressive function.

## Conclusions

**Fig. 9:**
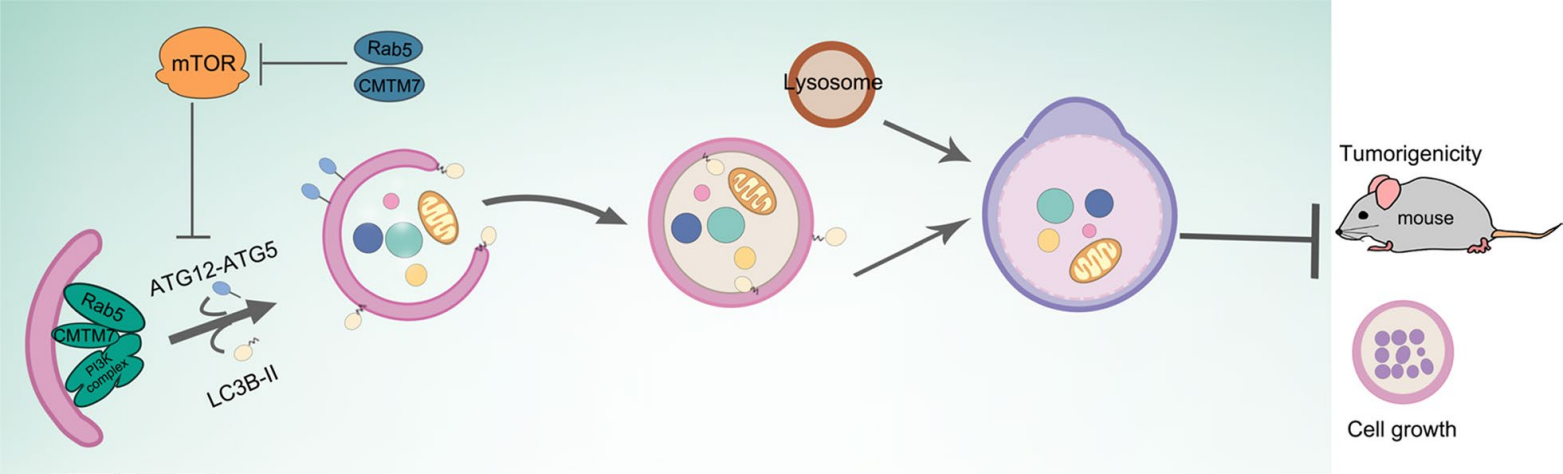
A model for the regulation of CMTM7 on autophagy and tumorigenicity. CMTM7 is mainly involved in the initiation of autophagosome formation through Rab5-mediated functional groups of ATG14L-Beclin1-VPS34 complex and mTOR signaling pathway, and plays a tumor-suppressive role by enhancing autophagy

In summary, our results suggested that CMTM7 is mainly involved in the initiation of autophagosome formation through Rab5-mediated functional groups of ATG14L-Beclin1-VPS34 complex and mTOR signaling pathway (Fig. 9). Both in vitro and in vivo experiments demonstrated that CMTM7 knockdown promotes tumor growth via impairing cell autophagy. These results provide a better understanding regarding the mechanism of CMTM7 on autophagy regulation and its tumor-suppressive role, resulting in more effective management of cancers by CMTM7 downregulation.

## Supplementary Information

**Additional file 1: Fig. S1.** Effect of CMTM7 knockdown on AMPKα phosphorylation. Western blot analysis of total and phosphorylation levels of AMPKα in control and CMTM7 knockdown A549 cells. β-actin was used as an internal standard.

## Data Availability

All data in our study are available upon request.

## Abbreviations

CMTM: Chemokine-like factor (CKLF)-like MARVEL transmembrane domain-containing family
MARVEL: MAL and related proteins for vesicle trafficking and membrane link
VPS34: Class III phosphoinositide 3-kinase vacuolar protein sorting 34
UVRAG: UV radiation resistance-associated gene
Atg14L: Autophagy-related 14-like protein
ATG: Autophagy associated gene
siRNA: Small interfering RNA
WIPI: WD-repeat domain phosphoinositide interacting
DAPI: Diamidino-phenyl-indole
DFCP1: Double FYVE-containing protein 1
DAPK1: Death-associated protein kinase-1
PTEN: The phosphatase that antagonizes PI3K
TSC1 and TSC2: Tuberous sclerosic complex 1 and 2
Bif-1: BAX-interacting factor 1
