# Supplementary figures and images for: CMTM7 as a novel molecule of ATG14L-Beclin1-VPS34 complex enhances autophagy by Rab5 to regulate tumorigenicity
Source: Cell Commun Signal. 2021 Jul 19;19:77. doi: 10.1186/s12964-021-00720-3 (PMC8287682; doi:10.1186/s12964-021-00720-3)

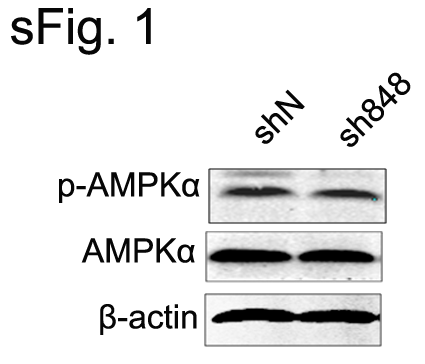

Supplement: Supplementary file 2 — Additional file 1: Fig. S1. Effect of CMTM7 knockdown on AMPKα phosphorylation. Western blot analysis of total and phosphorylation levels of AMPKα in control and CMTM7 knockdown A549 cells. β-actin was used as an internal standard. [file 12964_2021_720_MOESM2_ESM.tif]
